# Case Report: Cystinosis in a Chinese Child With a Novel CTNS Pathogenic Variant

**DOI:** 10.3389/fped.2022.860990

**Published:** 2022-04-15

**Authors:** Yu-Jia Guan, Yan-Nan Guo, Wen-Tao Peng, Li-Li Liu

**Affiliations:** ^1^Department of Nursing, West China Second University Hospital, Sichuan University, Chengdu, China; ^2^West China School of Nursing, Sichuan University, Chengdu, China; ^3^Key Laboratory of Birth Defects and Related Diseases of Women and Children (Sichuan University), Ministry of Education, Chengdu, China; ^4^Department of Pediatrics, West China Second University Hospital, Sichuan University, Chengdu, China

**Keywords:** cystinosis, CTNS gene mutation, Fanconi syndrome, renal tubular acidosis, genetics

## Abstract

**Objective:**

To report a rare case of cystinosis with a novel CTNS pathogenic variant in the Chinese population.

**Methods:**

Retrospective analysis of the clinical manifestations, laboratory results, and gene detection data of a child with cystinosis.

**Results:**

A Chinese Zang ethnic girl could not stand or walk until 3 years old, with additional symptoms including a loss of appetite. Since then, the girl gradually exhibited “X” leg, double wrist joints, a bilateral ankle deformity, and rickets. At the age of 9 years, the girl was hospitalized. Laboratory testing showed that her blood phosphorus, blood calcium and blood potassium levels were significantly decreased. At the same time, the girl's urine glucose and urine protein were positive, although her fasting blood glucose, glycosylated hemoglobin, and 75 g glucose tolerance were not significantly abnormal. Further, blood gas analysis showed metabolic acidosis. These symptoms corresponded to Fanconi syndrome. Gene analysis showed that there was a homozygous pathogenic variant c.140 ≤ 5G > A (p.?) in the CTNS gene, which was a small variation in the intron region. To our knowledge, this is the first report of the rare variant.

**Conclusion:**

Attention should be paid to the differential diagnosis of cystinosis by gene analysis in children whose clinical manifestations include exercise dysplasia, renal damage, or multiple organ damage (including bone, thyroid, etc) and who cannot be firmly diagnosed for the time being.

## Introduction

Cystinosis is an extremely rare autosomal recessive metabolic disease which is caused by the lack of L-cystine transporters on the lysosomal membrane resulted from the pathogenic variant of CTNS. This defect in lysosomal membrane transport leads to cystine accumulation, which eventually causes multiple organ dysfunction, including the eyes, kidneys, nerves, heart, and endocrine gland, amongst others. Until now, the majority of reported Cystinosis cases have occurred in Europe and the United States. The incidence of cystinosis in the general population is within the range of 1:260,000–1:115,000 (1, 2), which varies greatly in different regions. It should be noted that the relevant data in China are still incomplete.

In recent years, some progress has been made in the study of the disease in developed countries. However, relatively few reviews or case reports from China have been published (3–6). Furthermore, cases of cystinosis in children are particularly rare, which may be related to the low incidence of the disease and also the understanding of the disease. Timely findings, accurate diagnosis, and extensive reporting will be helpful to increase awareness of this disease. Meanwhile, early diagnosis and early treatment have an important impact on the prognosis of the disease, and can help to reduce or delay the occurrence of complications and prolong the patient's life. In this paper, the clinical manifestations, laboratory results, and gene detection data of a child with cystinosis diagnosed in West China Second Hospital of Sichuan University were reported (see below). The clinical characteristics, diagnosis and treatment strategies were discussed.

## Case Presentation

The patient was a 9-year-old girl who was the second child of non-consanguineous parents from the Zang ethnic minority group, Sichuan Province, China. Her mother was not exposed to or used alcohol, tobacco, or drugs during her pregnancy. She was carried full-term and had a normal vaginal delivery, although her birth weight and body length could not be provided by her family. She was breastfed after birth with supplementary foods added when appropriate. At the age of 1, the child began to crawl and sit by herself, which was not something her family paid particular attention to. However, by the age of 3, she could not stand or walk. Her mobility issues were accompanied by a poor diet, which mainly consisted of formula milk. The child's upper limb movement and cognitive and intellectual development were not significantly abnormal compared to children of the same age. Vitamin D and lysine were prescribed for her initial treatment in the outpatient department of a local hospital. At the age of 4 years, she could walk independently and appeared “X” leg. This patient was diagnosed with Fanconi syndrome, which based on renal tubular acidosis, vitamin D deficiency, growth retardation, normocytic anemia and the decreased serum phosphorus and calcium level during her first hospitalization, and diagnosed with bronchitis and hypocalcemia. Then she was treated with sodium dihydrogen phosphate, disodium hydrogen phosphate, sodium citrate + potassium citrate, vitamin D, and calcium.

After her symptoms were alleviated, it was suggested that she continue to take the above drugs after discharge and be followed up with the outpatient service. However, the child neither went to the clinic for follow-up on time, nor took medicine strictly according to the doctor's advice. Her family members admitted that they forgot occasionally and thought it was not such a serious problem. Their poor compliance may be related to distance from hospitals, lack of time, and family cultural background, concept and other reasons.

About 4 years later, at 8 years and 9 months old, the girl experienced fatigue and her hands were bent and claw-like. These symptoms were accompanied by pain, and at this time her family members independently decided to discontinue her medication. One month later, she presented with a paroxysmal dull ache in her hypogastrium, dizziness and headaches, and occasionally felt nauseous when eating greasy foods, soreness when exercised. She had occasional soft coughs, without polydipsia, urorrhagia, and so on.

At the age of 9, she was hospitalized in West China Second University Hospital of Sichuan University. The anthropometric evaluation showed that her stature (bodyweight 18 Kg, height 108 cm) was lower than the 3rd percentile of height and weight for individuals of the same age and sex, and also that her subcutaneous fat was thin. A Pectus carinatum brace was fitted. Upon testing, it was found that her blood phosphorus (1.07 mmol/L), blood calcium (1.72 mmol/L), and blood potassium (3.4 mmol/L) levels were significantly decreased. And it was found that her hemoglobin level (88 g/L) was decreased, while both of the level of Mean Corpuscular Volume (MCV) and the level of mean corpuscular hemoglobin (MCH) were within the normal range. This indicated that the patient can be diagnosed with normocytic anemia.

**Figure 1:**
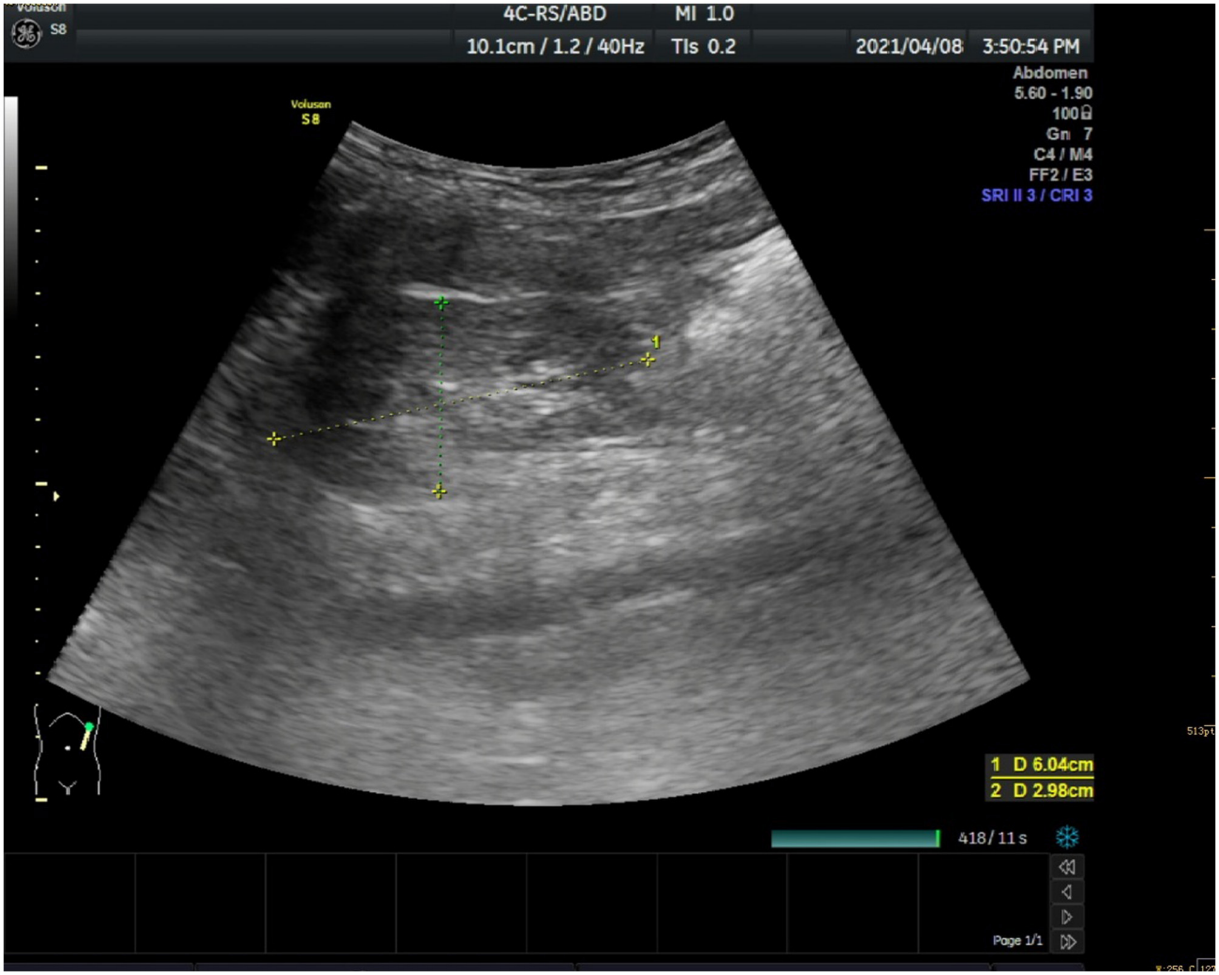
Renal ultrasound image.

Meanwhile, laboratory tests showed a decreased Vitamin D (both 25-OH-D and 1.25-OH-D) level (9.6 ng/ml), an increased alkaline phosphatase level (575 U/L), significantly increased blood urea nitrogen level (6.98 mmol/L) and creatine level (163 μmol/L). Urine sugar (3 +) and urine protein (2 +) were positive. Her levels of free carnitine (6.1 umol/L), citrulline (44.86 umol/L), glucose (75 g) tolerance, glycosylated hemoglobin, fasting blood glucose, and thyroid function were normal. Blood gas analysis showed metabolic acidosis and anion gap. Renal ultrasound image (Figure 1) showed slightly enhanced parenchymal echo in both kidneys.

The child's clinical symptoms included abnormal motor development for 6 years, with bone deformity, “X” legs, chicken chest, double wrist joints, bilateral ankle joint deformity, and other changes in her left bone and distal radius, ulna distal osteoporosis, including the decreased bone mineral density detected by bone densitometry of child via Quantitative Ultrasound Bone Densitometry (QUS), and left radius and distal ulna rickets. The child had rickets and hypophosphatemia in the past. A diagnosis of Fanconi syndrome was the preliminary clinical consideration. In addition, according to Schwartz formula, the Epidermal Growth Factor Receptor (eGFR) of the patient was 20.17 mL/(min 1.73 m^2^), indicating that the child was in chronic kidney diseases (stage IV) and did not meet the criteria for dialysis, so dialysis treatment was not performed for the time being. At the same time, we informed the patient's family that the patient was about to enter chronic kidney diseases (stage V), and preparations before dialysis should be made, including indwelling central venous catheterization for hemodialysis and peritoneal dialysis.

The patient suffered from renal tubular acidosis, hypokalemia, hypocalcemia and hypophosphatemia since childhood. The examination after admission showed that urine sugar (3 +) and urine protein (2 +) were positive, while glucose (75 g) tolerance, glycosylated hemoglobin and fasting blood glucose were normal. In addition, α1-microglobulin, β2-microglobulin and other proteins in urine that reflect the reabsorption function of renal tubules were increased. Therefore, the patient was considered to have renal tubular injury, which can lead to electrolyte disturbance, renal diabetes, and partial tubular proteinuria.

Meanwhile, considering the renal tubular injury, the decrease of serum calcium/phosphorus concentration/level and the increase of parathyroxine levels (945.8 pg/ml), the secondary hyperparathyropathy could be diagnosed. Furthermore, the girl had abdominal pain for more than 3 months prior to hospitalization. A CT scan showed slightly blurred mesenteric fat space, multiple enlarged lymph nodes in the abdominal cavity were identified, thus mesenteric lymphadenitis was considered.

During hospitalization, the girl appeared tetany repeatedly, manifested as flexion and rigidity of the limbs as well as chicken claw-like hands accompanied by pain, and the lowest blood calcium level was 1.68 mmol/L. Calcium carbonate was then supplemented intravenously and oral calcium zinc gluconate and ossified triol were added. As citrate would combine with free calcium ions, which could further decrease blood calcium levels, the administration of citrate was stopped.

The girl underwent ophthalmologic examination in West China Fourth Hospital of Sichuan University. The conjunctiva, cornea, iris, lens, retina, intraocular pressure, and diopter were normal, no binocular corneal crystalline was found, and there was no visual field defect.

On the premise of informed consent, the whole exome sequencing (WES) of this girl was conducted. We decided to perform WES analysis because the initial onset age of the patient was early, accompanied by multiple organ system damage, and the treatment effect of oral sodium and potassium citrate mixture for renal tubular acidosis was not good. Meanwhile, the renal function of this patient showed progressive decline. The progression of renal tubular dysfunction, followed by glomerular involvement and renal decompensation, led us to strongly suspect that the patient had a mono-genetic inherited related renal tubule dysfunction.

**Figure 2:**
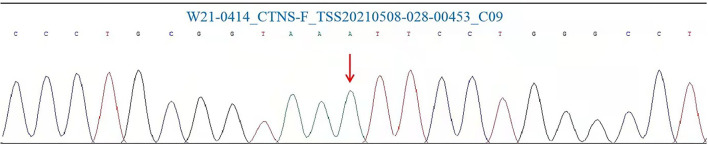
CTNS mutation Sanger analysis results for the patient involved in this study. Polymerase chain reaction (PCR) and Sanger was performed in our test.

Gene analysis showed that there was a homozygous pathogenic variant c.140 ≤ 5G > A (p.?) in the patient's CTNS gene (Figure 2). This was a small variation in the intron region, found for the first time. Notably, such a variant has not been reported in the literature and is rare in the population. Combined with the results of genetic examination, the diagnosis of cystinosis was made.

Although the child was in a serious condition, her parents asked for her to be discharged after 6 days of hospitalization. Research shows that hypophosphatemia and hypocalcemia in Fanconi syndrome and proximal renal tubular acidosis can be exacerbated by vitamin D deficiency, and the resulting tetany and obvious bone pain. And the above clinical symptoms can be effectively alleviated by the treatment with sodium hydrogen phosphate and sodium phosphate disodium buffer solution and active vitamin D.

After discharge, the parents were guided to supervise the girl to use drugs according to doctor's prescriptions (to take orally sodium dihydrogen phosphate, disodium hydrogen phosphate, lysine inositol vitamin B12, lysine calcium hydrogen phosphate tablets, calcitriol soft capsules, ferrous succinate tablets and vitamin C, and to recheck regularly).

The girl was asked to limit the intake of high protein foods, and return to the hospital if there was any discomfort. Twenty-eight days after the discharge, her family received the gene report results and were advised to carry out the related gene detection. Unfortunately, her family refused to carry out genetic testing. We used network follow-up to remind her family members to take her to the local hospital for urine review to monitor kidney function, but the patient did not follow up strictly according to the doctor's advice.

## Discussion

Up to date, most of the reported cases of cystinosis have occurred in Europe and the United States, with much less cases of cystinosis reported in Asia. Specifically, one case in Japan (7), 6 cases in Thailand (4 families involved) (8), 2 cases in India (9), and 21 cases in China have been reported (Table 1) (3, 5, 6, 10–13). The Chinese cases included 10 Mainland families (including 13 patients with CTNS homozygous mistranslation variant N323K, c.681 G > A(p.E227E), c.477C > G (p.S159R), c.274C > T (p.Q92X), c.680A > T (p.E227V) and CTNS pathogenic variant of (IVS6+1, del G and IVS8-1, and del GT), or with a deletion of c.18_21del GACT, (p.T7FfsX7)), and 5 Taiwanese families (including 8 patients with CTNS homozygous mistranslation variant N323K, 753G > A(W138X), exon 11 (IVS11+2T > C), 1178A-G (K280R) or mutation 57-kb deletion cystinosis).

**Table 1:** Clinical and molecular characteristics of Chinese patients with cystinosis.

**Study**	**Case**	**Nationality**	**Age of onset**	**Age of diagnosis**	**Mutation site**	**Cystinosis subtypes**	**Extra renal symptoms**	**Renal impailment**	**Treatment**
Du et al. (3)	1	–	1 year	13 years	c. 969C > G, p. 323N > K	Nephropathic	Growth retardation, rickets, hypothyroidism	Tubular dysfunction, including glycosuria, urine protein, Amino acid urine, high phosphate urine	Taken neutral phosphorus mixture, citric acid mixture, sodium bicarbonate tablets, calcitriol, levothyroxine sodium. Limit the intake of high protein foods
	2	–	1 year	6 years	c. 969C > G, p. 323N > K	Nephropathic	Growth retardation, rickets	Tubular dysfunction, including glycosuria, urine protein, Amino acid urine, high phosphate urine	Taken neutral phosphorus mixture, citric acid mixture, sodium bicarbonate tablets, and calcitriol limit the intake of high protein food
Li et al. (10)	1	–	1 year	12 years	Homozygous c.969C > G, (p.N323K)	Nephropathic	Binocular corneal crystalline, growth retardation, rickets, hypothyroidism, metabolic acidosis	Glycosuria, urine protein, abdominal ultrasound showed renal damage	Citrate mixtures, phosphate supplements, oral calcium agents and calcitriol, GH treatment, Thyroid stimulating hormone, sodium levothyroxine tablets, calcium carbonate, sodium bicarbonate, gonadotropin releasing hormone. When this patient became 14 years old (40 kg), he was able to access cysteamine with international help. However, the patient suffered from severe pain in the legs and massive acne on both sides of the nose within 1 month of cysteamine treatment, which led to the halt of the treatment
	2	–	6 months	5 years	Homozygous c.969C > G, (p.N323K)	Nephropathic	Binocular corneal crystalline, growth retardation, rickets, hypothyroidism	Glycosuria, urine protein	Patient 2 was the brother of patient 1, and his symptoms were similar to patient 1 and he received comparable treatment as mentioned earlier
	3	–	1 year	4 years	c.18_21del GACT, (p.T7FfsX7); c.477C > G (p.S159R)	Nephropathic	Binocular corneal crystalline, growth retardation, rickets	Normal	Treated with alternative therapies to maintain the electrolyte levels, but none of them received either cysteamine or GH treatment
	4	–	1 year	6 years	Homozygous c.18_21del GACT, (p.T7FfsX7)	Nephropathic	Binocular corneal crystalline, growth retardation, rickets	Could not acquire	Treated with alternative therapies to maintain the electrolyte levels, but none of them received either cysteamine or GH treatment
	5	–	3 years	5 years	c.274C > T (p.Q92X)a; c.680A > T (p.E227V)	Intermediate	Binocular corneal crystalline, growth retardation, rickets	Chronic renal insufficiency (stage III)	Treated with alternative therapies to maintain the electrolyte levels, but none of them received either cysteamine or GH treatment
	6	–	9 months	5 years	c.18_21del GACT, (p.T7FfsX7); c.600_700del GT, (p.S234LfsX61)	Nephropathic	Binocular corneal crystalline, growth retardation, RicketsH6:H9	Kidney failure (ESRD) and waiting for a kidney transplant	Treated with alternative therapies to maintain the electrolyte levels, but none of them received either cysteamine or GH treatment
Ling et al. (6)		–	–	4 years	c.681 G>A (p.E227E)	–	Binocular corneal crystalline, growth retardation, rickets, metabolic acidosis	Polyuria, glycosuria, urine protein	It is only mentioned that this patient did not comply with cysteamine treatment
Jiang et al. (11)		–	1 year	4 years	–	Ocular non-nephropathic cystinosis	Binocular corneal crystalline, growth retardation, rickets	Glycosuri, urine protein, Amino acid urine	Correct acidosis, supplement potassium, sodium and calcium, and topical eye drops of cysteine hydrochloride can effectively remove corneal crystallization
Yang et al. (5)	1	Han	7 months	7 years and 6 months	CTNS (IVS6+1, del G and IVS8-1, and del GT)	–	Binocular corneal crystalline, growth retardation, rickets, metabolic acidosis (severe), moderate anemia, secondary carnitine lack	Polydipsia and polyuria, Systemic amino acid uria, Fanconi syndrome	Lactose-free diet, supplemented with carnitine, VitB1, and VitB12
	2	Han	7 months	3 years and 4 months	CTNS (IVS6+1, del G and IVS8-1, and del GT).	–	Growth retardation, rickets, metabolic acidosis, hypokalemia, iron deficiency anemia, secondary carnitine deficiency	Glycosuria, urine protein, polydipsia and polyuria, renal Fanconi syndrome	Could not acquire
Chuang et al. (12)	1	–	–	5 years	–	–	Binocular corneal crystalline	Kidney failure (ESRD) and received allografts	Began cysteamine treatment until transplantation at ages 13.4, and delivered a girl without complication during gestation, and her renal function also remained good
	2	–	–	9 years	–	–	Binocular corneal crystalline	Kidney failure (ESRD) and received allografts	Began cysteamine treatment until transplantation at ages 19.8 and 26.4 years. Obstructive nephropathy with progressive graft failure at age 26.4 years and was treated for vulvar condyloma and carcinoma *in situ* of cervix
Thoene et al. (13)	1	–	8 years and 1 months	–	1308C > G (N323K)	Nephropathic	Binocular corneal crystalline	Proteinuria, At age 12 her creatinine clearance had fallen to 67 mL/min/1.73 m^2^ and her proteinuria had increased to 3.2 g/day	–
	2	–	5 years and 8 months	–	1308C > G (N323K)	Nephropathic	Binocular corneal crystalline	Proteinuria, At age 12 9/12 her daily protein excretion is 2.7 g and creatinine clearance 18 mL/min/1.73 m^2^	–
	3	Unknown	7 years	13 years	753G > A (W138X), exon 11 (IVS11+2T > C)	Intermediate	Binocular corneal crystalline	Kidney failure (ESRD)	Renal transplant at age 15
	4		5 years	11 years	753G > A (W138X), exon 11 (IVS11+2T > C)	Intermediate	Binocular corneal crystalline	Kidney failure (ESRD)	Renal transplant at age 14
	5		16 years	18 years	753G > A (W138X), exon 11 (IVS11+2T > C)	Intermediate	Binocular corneal crystalline, moderate hypertension, minimal photophobia, thyroid gland is slightly enlarged	Proteinuria, creatinine clearance has declined to 42 mL/min/1.73 m^2^	–
	6		12 years	25 years	57-kb deletion, 1178A-G (K280R)	Intermediate	Binocular corneal crystalline	ERSD	Hemodialysis and renal transplant at age 30 at age 43, the serum creatinine was 1.4 mg/dL
Ma et al. (4)	1	–	2 years	–	c.696C > G(p.323 N > K)	Nephropathic	Binocular corneal crystalline, growth retardation, rickets	Tubular dysfunction	It is only mentioned that supportive treatment and specific treatment can be used

At present, the known types of gene pathogenic variant include insertion, deletion, repetition, translocation, point variant, splice site variant, promoter variant, and genome rearrangement of CTNS gene sites. Here we report a new homozygous variant of the CTNS gene. The pathogenic variant site was lntron4 position on chr17:3550821 chromosome, and the protein level of reference transcript was NM 004937.3: c.140+5G > A (p.?). The variation is a small variation in the intron region, which has not been reported in the literature as this variant is rare in the population. The variation cannot be found in the ESP6500siv2 ALL, 1000g2015aug ALL, EXAC, gnomAD, and dbSNP147 databases. Unfortunately, the family members of the child refused to undergo genetic testing, and as such, we could not determine whether other members of the family had similar variations.

Also, among the cases reported in China, only Yong-jia Yang's report clarified ethnic groups of cystinosis patients, in which a Chinese Han family was affected.

Compared with adults, there are very few reports on cystinosis in children in China. However, in recent years, scholars are increasingly paying attention to cystinosis in children. Based on the duration of presented symptoms and the severity of renal involvement, Cystinosis can be divided into infant type (also known as kidney type), juvenile type (also known as intermediate type), and ophthalmopathy type, of which infant type is the most common and most serious, typically characterized by kidney involvement as the first symptom (14). In our report, the patient belongs to infant type, manifested as involvement of multiple organs such as kidney, bone, and thyroid. The girl began to develop symptoms at the age of 3 and was first diagnosed with Fanconi syndrome at the age of 4. At the age of 9, she was hospitalized due to the aggravation of the symptoms. At this time, following gene detection, she was diagnosed with cystinosis and chronic kidney disease. It took 5 years from the initial visit to the final diagnosis for this case. This may be related to the low medical cooperation of the patient's family members, characterized by irregular medical treatment, poor medication compliance, etc. There are also other factors, including the rarity of the cases, economic conditions, limit of diagnosis, and treatment technologies as well as the concept of medical behavior of the patient and her family members. In recent years, with the development of economy and the progress of diagnosis and treatment technologies, gene detection technology has gradually become popularized in China, which has greatly enriched the means of diagnosis and treatment and improved the level of treatment.

At present, on the premise of combining clinical manifestations and laboratory results, there are three methods to assist in the diagnosis of suspected cystinosis patients: (1) Using mass spectrometer liquid chromatography to detect the level of cystine in blood leukocytes. This method has high sensitivity and specificity, and can monitor the therapeutic effect. However, as the requirements for instruments and testers are high, it cannot be easily conducted in a clinic. (2) Most patients can be diagnosed by gene detection, although the price is relatively high. (3) Using a slit lamp to detect characteristic corneal cystine crystallization is simple and economical; however, the scope of application is narrow: it is only suitable for patients with eye symptoms and cannot monitor the therapeutic effect.

Therefore, the corresponding medical testing methods can be selected based on the local medical conditions and the clinical manifestation type of the patient in question. At present, some cases of cystinosis in China have been analyzed by gene analysis, with the number of such cases increasing in recent years (3, 5, 6, 10–13). Gene diagnosis is beneficial to the diagnosis and analysis of cystinosis, and it is also an area for development in the future (15).

Some studies have pointed out that, the cases diagnosed may only be part of the actual cases because of the difficulty of diagnosis, such that some patients may not be able to be diagnosed (16). If patients with cystinosis cannot be diagnosed in time, they will not be treated in time, meanwhile the genetic information and clinical data of this population will be missing. From this case, it can be seen that the treatment compliance of the child and her families is not high. Thus, medical and nursing staff need to work to improve the treatment compliance of children and their families.

Health outcomes can be improved through health education and the improvement of drug research and treatment methods, such as developing sustained-release capsules to reduce the frequency of drug taking (17), and informing children and their families of the serious consequences of drug withdrawal through real cases examples.

At present, mercaptamine bitartrate, a drug that can specifically reduce cystine in lysosomes, is the preferred treatment for nephropathic cystinosis. The drug was transported to lysozyme by lysosomal membrane PQLC2 vector by forming cysteamine cysteine binding molecule with cystine in lysozyme. Early medication can effectively improve the growth and development, delay the occurrence of end-stage renal disease, but cannot prevent the progress of the disease. A new cysteamine preparation with delayed release simplifies the administration schedule, but still cannot cure cystinosis disease. Mercaptamine bitartrate is widely used in developed countries, but has not yet been introduced into China, which limits our research on this disease.

In the future, therapeutic research may be carried out based on CTNS-carrying stem cell transplantation, which has been successfully carried out in mouse model. Hematopoietic stem cell transplantation and gene editing technology are possible choices to cure the diseases (18).

As our understanding has developed, cystinosis has gradually become a treatable rare disease. Going forward, it is very important to improve the awareness by timely reporting of the disease and to adopt the behavior of actively seeking medical treatment. Early detection and early treatment can reduce and delay the occurrence of complications and improve the prognosis of patients with cystinosis.

## Data Availability Statement

The datasets for this article are not publicly available due to concerns regarding participant/patient anonymity. Requests to access the datasets should be directed to the corresponding author.

## Ethics Statement

Written informed consent was obtained from the individual(s), and minor(s)' legal guardian/next of kin, for the publication of any potentially identifiable images or data included in this article.

## Author Contributions

All authors listed have made a substantial, direct, and intellectual contribution to the work and approved it for publication.

## Funding

This article was supported by the fund of Science and Technology Bureau of Sichuan Province (Grant No. 2019YFS0240).

## Conflict of Interest

The authors declare that the research was conducted in the absence of any commercial or financial relationships that could be construed as a potential conflict of interest.

## Publisher's Note

All claims expressed in this article are solely those of the authors and do not necessarily represent those of their affiliated organizations, or those of the publisher, the editors and the reviewers. Any product that may be evaluated in this article, or claim that may be made by its manufacturer, is not guaranteed or endorsed by the publisher.
